# Dengue virus: pathogenesis and potential for small molecule inhibitors

**DOI:** 10.1042/BSR20240134

**Published:** 2024-08-14

**Authors:** Navya Chauhan, Kishan Kumar Gaur, Tejeswara Rao Asuru, Prasenjit Guchhait

**Affiliations:** Regional Centre for Biotechnology, National Capital Region Biotech Science Cluster, Faridabad, India

**Keywords:** Antiviral, Dengue, Therapeutics

## Abstract

Dengue, caused by dengue virus (DENV), is now endemic in nearly 100 countries and infection incidence is reported in another 30 countries. Yearly an estimated 400 million cases and 2200 deaths are reported. Effective vaccines against DENV are limited and there has been significant focus on the development of effective antiviral against the disease. The World Health Organization has initiated research programs to prioritize the development and optimization of antiviral agents against several viruses including *Flaviviridae*. A significant effort has been taken by the researchers to develop effective antivirals against DENV. Several potential small-molecule inhibitors like efavirenz, tipranavir and dasabuvir have been tested against envelope and non-structural proteins of DENV, and are in clinical trials around the world. We recently developed one small molecule, namely 7D, targeting the host PF4-CXCR3 axis. 7D inhibited all 4 serotypes of DENV *in vitro* and specifically DENV2 infection in two different mice models. Although the development of dengue vaccines remains a high priority, antibody cross reactivity among the serotypes and resulting antibody-dependent enhancement (ADE) of infection are major concerns that have limited the development of effective vaccine against DENV. Therefore, there has been a significant emphasis on the development of antiviral drugs against dengue. This review article describes the rescue effects of some of the small molecule inhibitors to viral/host factors associated with DENV pathogenesis.

## Introduction

One of the rapidly emerging viral diseases dengue is now endemic to more than 100 countries across tropical and subtropical regions of the globe including India [1]. Each year an estimated 400 million cases and 2200 deaths are reported [2]. Throughout the period from 2000 up to 2019, the World Health Organization (WHO) reported a 10-fold rise in cases globally, from 500,000 to 5.2 million. The year 2019 was an unprecedented peak with reported incidents covering 129 countries. While the COVID-19 pandemic and reduced reporting rates caused a slight drop in cases between 2020 and 2022, there has been an increase in dengue infections worldwide since 2023 marked by notable multiplication of outbreaks in both numbers and magnitude concurrently occurring beyond dengue-free areas before.

Dengue is caused by dengue virus (DENV), a single positive-stranded RNA virus of the *Flaviviridae* family, mainly transmitted by *Aedes aegypti* trailed by other mosquitoes belonging to genus *Aedes* [3]. DENV genome encodes three structural proteins (Capsid protein C, Membrane protein M and Envelope Protein E) and seven non-structural proteins (NS1, NS2a, NS2b, NS3, NS4a, NS4b, and NS5) [1]. The RNA genome is divided into three parts: 5′ UTR region (untranslated region), ORF (open reading frame), and 3′ UTR region [4]. DENV is composed of 10,723 nucleotides (approximately 11 kb), which are used to encode larger polyprotein precursors containing ∼3391 amino acid residues [5]. The DENV life cycle is divided into various stages, including viral entry, fusion and disassembly, viral genome replication, viral protein translation and processing, assembly, maturation, budding, and release. The process begins when the virus binds to receptors on a susceptible host cell, causing receptor-dependent endocytosis [6]. The DENV E protein binds to the host cell via interacting with a number of cellular factors present on the target cells, including dendritic cell (DC)-specific intercellular adhesion molecule-3-grabbing non-integrin (DC-SIGN), the mannose receptor, heparan sulfate, FC receptor, and others. DENV uses clathrin-mediated endocytosis to enter the intended cell, enabling the endosome membrane to fuse with DENV, releasing the RNA genome [7]. Viral proteins are produced by the cleavage of a polyprotein resulting from the translation of positive-sense viral RNA by both host and viral proteases. In parallel, an intermediate negative RNA template is created using the positive-sense viral RNA and is utilized as a template for additional genomic replication. During the maturation phase, the replicated genomes and generated viral proteins are put together into virions inside the endoplasmic reticulum (ER). The host enzyme furin cleaves the prM protein into membrane (M) protein as the virions enter the Golgi vesicles, promoting viral maturation. Ultimately, the mature virions are released from the cell via exocytosis [6]. When the virus replicates both the structural and non-structural proteins are transcribed and translated to be available for intracellular antigen processing pathways. These various roles are performed by these NS proteins. Interacting with NS4A/B facilitates replication of the virus by promoting viral replication through binding with NS1 while helicase/protease functions as a protease/helicase on its own [8]. Dengue shock syndrome (DSS) and dengue haemorrhagic fever (DHF) represent critical manifestations. The DENV comprises four distinct serotypes DENV1, DENV2, DENV3, and DENV4. Despite similarities in their amino acid sequences, ranging from 70% to 80%, each serotype poses unique challenges. Notably, individuals previously exposed to one serotype face an elevated risk of severe dengue if infected by another serotype, highlighting the importance of cross-immunity [9]. This is because the sub-neutralizing cross-reactive antibodies opsonize virus particles, facilitating infection of mononuclear phagocytes via Fc-receptor (FcR), a phenomenon called antibody-dependent enhancement (ADE) of infection [10–12]. In 2009, the WHO issued a guideline dividing symptomatic cases into two subgroups: non-severe and severe dengue, further dividing non-severe into symptomatic and symptomatic. The clinical symptoms of febrile illness include low platelet counts, tiredness, irritability, bleeding from body openings, clinical fluid accumulation in the body or legs, tummy pain or tenderness as well as large liver. The severe dengue is characterized by significant organ damage, substantial plasma leakage and haemorrhage, resulting in thrombocytopenia, coagulation abnormalities, vasculopathy, or disseminated intravascular coagulation [13].

**Figure 1:**
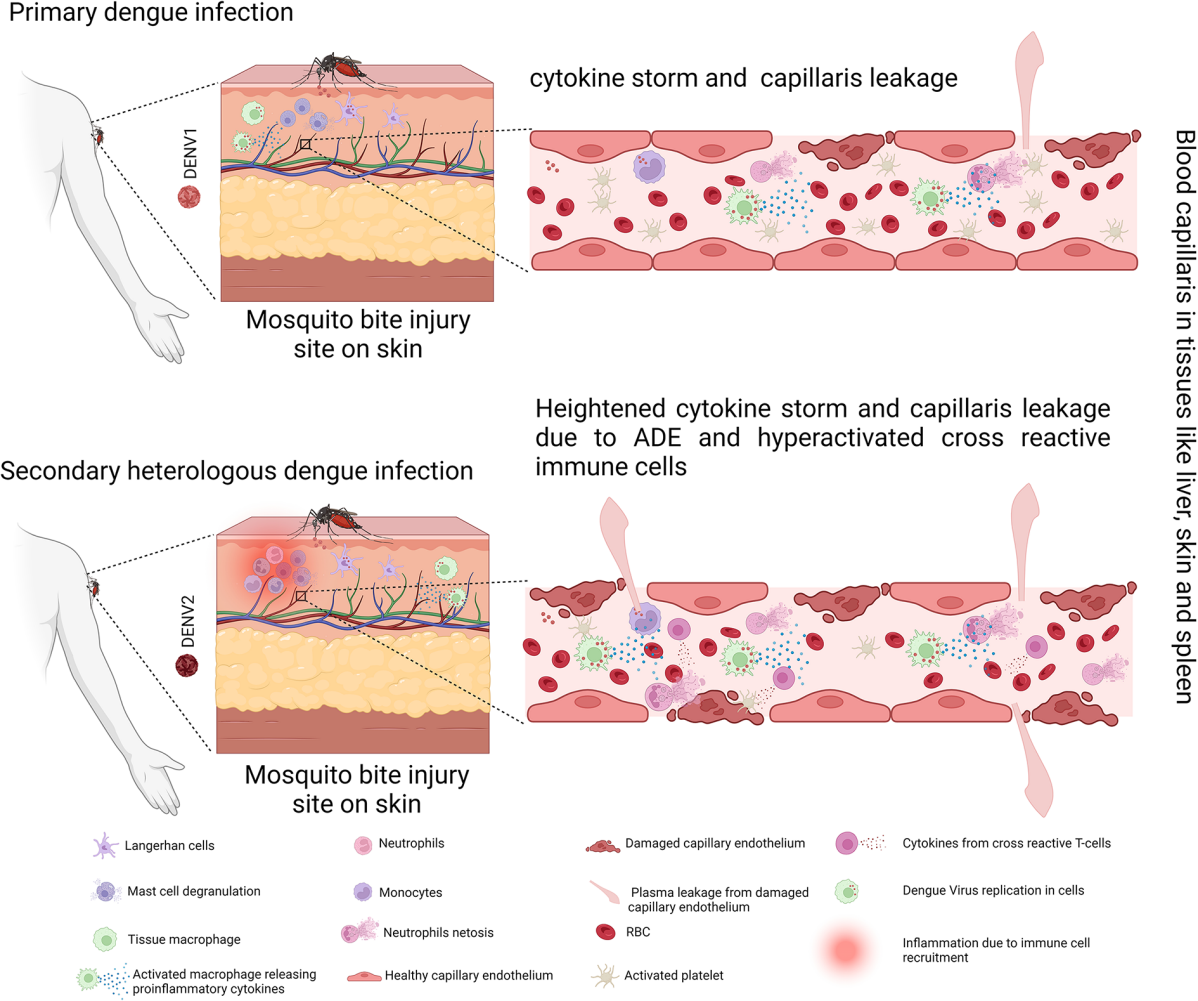
Dengue pathogenesis in primary and heterologous secondary infection Created with Biorender.com.

This review article elucidates the therapeutic impact of specific small molecule inhibitors on viral and host factors implicated in DENV pathogenesis. Small molecules, defined as low molecular weight organic compounds typically less than 900 Daltons, can regulate biological processes and are widely used in medicinal chemistry to modulate protein functions and pathways due to their ability to easily diffuse across cell membranes. In the context of DENV, these small molecules are being explored as potential antiviral agents. Researchers are investigating these compounds for their ability to inhibit various stages of the DENV life cycle, including entry into host cells, replication, and assembly. By targeting specific viral proteins or host factors essential for viral replication, small molecules could potentially serve as effective treatments to reduce the severity and spread of dengue infections [146]. Antibody cross reactivity among the serotypes and resulting ADE of infection are major concerns that have limited the development of effective vaccines against DENV and there has been, therefore, a significant emphasis on the development of antiviral drugs against dengue. Current research focuses on the development of effective antivirals that can be prescribed early in the course of infection that would prevent transmission of the viruses. The WHO has initiated research programs to prioritize the development and optimization of antiviral agents against several viruses including *Flaviviridae*. Unfortunately, at the current scenario, there are no approved antivirals against dengue [14]. Many antiviral compounds that inhibit DENV replication have been identified *in vivo* and *in vitro* by several groups of researchers [15]. Specific inhibitors targeting the viral envelope [16], NS4B [17], methyl transferase [18], protease [19], and host enzymes such as ER glucosidase [20] have been identified and demonstrated antiviral activity. There is presently no licensed antiviral therapy for DENV infection in humans, despite their apparent efficacy in preventing DENV replication [21]. This review article highlights the urgent need for effective interventions and treatment options for dengue fever (Figure 1).

## Pathogenesis

During mosquito feeding on humans, DENV is apparently injected into the bloodstream, with spill over in the epidermis and dermis, leading in infection of young Langerhans cells (epiderma DCs) and keratinocytes Following infection, cells that have been infected migrate from the initial site of infection to the lymph nodes. Monocytes and macrophages are attracted to the lymph nodes and hence become susceptible to infection. This increases the infection and spreads the virus throughout the lymphatic system. The term first ‘viremia’ refers to the initial phase of viral infection, during which the virus enters the bloodstream and begins disseminating. This stage involves the infection of numerous mononuclear cells, including monocytes from the bloodstream, myeloid DCs, and macrophages in the spleen and liver. During the initial viral infection in dengue, the virus spreads quickly across the host’s circulatory system, resulting in systemic dissemination. This phase is crucial for infection establishment because the virus gains access to many tissues and organs, allowing for more replication and amplification. Infected mononuclear cells, especially DCs and macrophages, play an important role in delivering the virus to lymphoid organs, where it can evade the host’s immune response and spread. The intensity and duration of initial viremia are major predictors of illness severity and can have an impact on clinical outcomes in dengue patients. This stage must be managed effectively in order to control the infection’s spread and progression [22,23].

Even in secondary infections, severe dengue is infrequent, occurring in just 0.5% to 1% of cases, resulting in severe syndromes. In which antibodies produced during the initial DENV infection bind to but do not neutralize the different DENV serotype. It has been proposed that binding an antibody to a virus of a different serotype allows the immune cells like macrophages and monocytes, potentially up taking more viruses, leading to increased virus production, viremia, and pathogenesis [24]. These antibodies let the virus enter cells with FcγR receptors, which increases viral levels and worsens the sickness [25]. Furthermore, FcγR-mediated DENV infection effectively suppresses the host’s antiviral innate immune response, leading to increased intracellular replication [26]. Some of these mechanisms include antibody-dependent enhancement, cell-mediated pathogenesis, the cytokine storm phenomenon, an individual’s genetic background, virus strain differences, virus levels circulating in individuals during the acute phase, and the infected individual’s nutritional status [27].“Summarized in (Figure 1)”.

Researchers have developed some potential small-molecule inhibitors like efavirenz, tipranavir and dasabuvir against *Flaviviruses* [28]. The efavirenz was developed to target a non-nucleoside reverse transcriptase inhibitor (NNRTI) of HIV infection. Recent research suggests that efavirenz may also have antiviral effect by blocking the DENV NS5 protein [29]. Tipranavir is a protease inhibitor authorized for the treatment of HIV. Interestingly, tipranavir exhibits antiviral by targeting NS2B-NS3 protease of DENV [30]. The small molecule AZD0530 and Dasatinib have been indicated to reduce DENV2 RNA replication, resulting in decreased steady-state viral RNA accumulation within cells. In addition, research has shown that depleting Fyn kinase using RNA interference (RNAi) resulted in significant suppression of DENV2. Fyn kinase activates SYK protein, which in turn enhances PLC gamma activity. This activity results in the production of DAG and IP3. IP3 increases calcium levels, while DAG activates PKC, leading to the production of NF-κB. The activation of NF-κB subsequently releases cytokines and chemokines, reducing viral infection [31]. Research on JNJ-1802, an effective DENV inhibitor, successfully targets the DENV non-structural protein NS4B and disrupts its interaction with NS3. NS4B is crucial for evading the host's antiviral response by inhibiting the activation of STAT1 and interferon-stimulated genes (ISGs), i.e. IRF1, IRF2, IRF 7, IRF9, ISG15, ISG20, RIG-1, etc., decreasing the host cell's antiviral defence mechanisms [32]. Another study related to small molecule inhibitor of dengue has shown that DHBTs dihydrobenzo thiepines (tricyclic small-molecule) significantly restrict DENV2 infection by altering the ERK signalling pathway via DRD4 regulation, resulting in a reduction in viral replication. Antagonism of DRD4 and subsequent downstream phosphorylation of epidermal growth factor receptor (EGFR)-related kinase (ERK) were discovered to have a negative impact on DENV infection, and blocking signalling through this network was confirmed as the mechanism of anti-DENV activity for this class of compounds [10]. Our laboratory research shows that PF4 promotes p38 MAPK phosphorylation and prevents STAT-2 and IRF-9, in turn decreases IFN-α synthesis in monocytes following DENV2 infection. Importantly, this route was reversed by either anti-PF4 antibody or the PF4 receptor CXCR3 antagonist AMG487, which allowed IFN-α secretion in infected monocytes [33]. This review article describes the rescue effects of small molecules antagonist to several factors associated with DENV pathogenesis.

## Small molecules targeting DENV proteins

**Figure 2:**
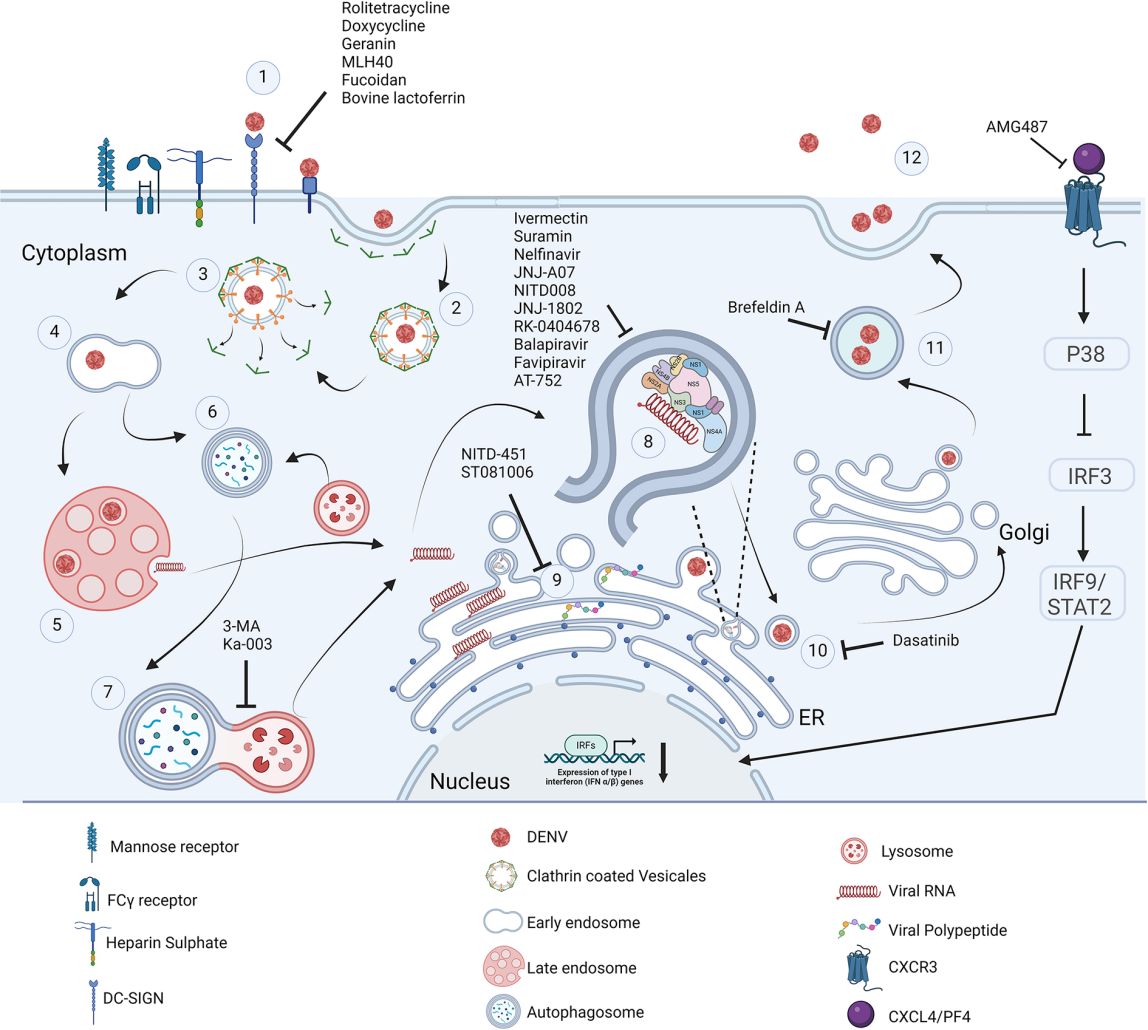
Dengue virus replication cycle in host immune cell It begins with (1) Attachment of virus to host receptor via; (2) clathrin mediated endocytosis further; (3) Disassembly of clathrin coated virus containing vesicles; (4) Forming early endosomes; (5) Maturing into late endosomes and fusion leading to disassembly of virus and release of viral RNA due to low pH; (6) Early endosomes fuse with autophagosomes to facilitate successful replication of DENV further; (7) Fuse with lysosomes for degradation; (8) Viral RNA in cytoplasm replicates in the replication complexes; (9) Translates into virus polypeptide in endoplasmic reticulum (ER); (10,11) Virus assembly and maturation takes place in the golgi apparatus; and further (12) egress from the cells. Created with Biorender.com.

The recent COVID-19 pandemic has again highlighted the importance of developing antiviral to combat such diseases. Effective antiviral can be prescribed early in the course of infection to prevent transmission of the viruses. The World Health Organization has initiated research programs to prioritize the development and optimization of antiviral agents against several viruses including *Flaviviridae*. Small molecules which target the specific viral protein and exerts its antiviral properties are called as direct acting antivirals [34]. They will have promising antiviral activity and low toxicity in comparison to host directed small molecules. Development of resistance is the major drawback in developing these direct acting antivirals [35]. Development of such small molecules need the approach of structurally understanding the different proteins encoded by the DENV genome. In DENV, Envelope (structural protein), NS1, NS3 and NS5 (Non-structural proteins) are well studied as target proteins due to their functional activity [36] (Summarized in Figure 2).

### NS1

Antiviral small molecules towards NS1 are not that common but studies have shown that peptides which selectively bound DENV NS1 and inhibited all DENV serotypes except DENV3 effectively in human cell lines. However, the higher concentrations of these peptides were found to be cytotoxic [37]. Another study has found that miRNA let-7a inhibited all the serotypes of DENV by targeting a highly conserved sequence of NS1. Studies also found that extracts from honeysuckle plants increase the expression of miRNA let-7a both *in vitro* and *in vivo*. Mice treated with these extracts were rescued from dengue infection and pathogenesis [38].

### NS2B/NS3

DENV NS3 protein is the major antiviral target due to its varied functions through multiple domains. Its major enzymatic activities include 5′-RNA triphosphatase, helicase, N-terminal protease domain cleaving the viral polyprotein and its C-terminal RNA helicase domain aiding in viral RNA replication and synthesis. NS2B acts as a cofactor for NS3 for proper folding and activity. Protegrin-1, disulfide cyclic peptide, and retrocyclin-1 show antiviral activity by inhibiting the NS2B-NS3 protease [39,40]. Nelfinavir, a repurposed drug, was able to selectively bind to this protease and inhibit the DENV infection.

The helicase domain of NS3 lacks binding pockets making it difficult to the develop drugs towards NS2B-NS3 protease. Some drugs have been shownto inhibit the NS3 helicase domain, Ivermectin is one such antiviral that inhibits NS3 helicase activity by non-competitively binding to the helicase domain [41]. ST-610, a benzoxazole compound and suramin a drug used to treat African sleeping sickness are found to non-competitively inhibiting NS3 helicase [63,64].

### NS3/NS4A and B

Dengue NS4 comprises two full-length membrane proteins binding to the ER membrane replication complexes [137]. The NS4A as cofactor for NS3 act as a signal sequence to translocate NS4B into ER lumen. NS4B is known to inhibit STAT1 phosphorylation by blocking the signalling cascade initiated by IFNα/β [42,43]. Studies have shown that AM404 a metabolite generated from paracetamol metabolism is having an inhibitory effect on DENV replication by inhibiting NS4B. Mutations in NS4B are shown to render this metabolites effectivity indicating that this metabolite bind to NS4B to exert its activity [44]. Compound-1a which is further improved to Compound-14a, a spiropyrazolopyridone compound is found to inhibit DENV infection in AG129 mice. NITD-618, a nucleoside analog, is also found to be an inhibitor of NS4B. These compounds are confirmed to be the inhibitors of DENV NS4B through mutation screening assays [17,45]. JNJ-64281802 is another NS4B inhibitor that showed antiviral properties *in vitro*. Its analog JNJ-A07 was shown to be effective in decreasing the DENV load and pathogenesis in AG129 mice [97,129]. Both of these small molecules block the NS3-NS4 complex which is essential for the formation DENV replication complex.

### NS5

NS5 is the largest dengue non-structural protein with various biological and enzymatic functions. NS5 has an N-terminal methyltransferase (MTase) domain for 5′ RNA cap synthesis and methylation, and a C-terminal RNA-dependent RNA polymerase (RdRp) domain for viral RNA synthesis. NS5 is found to be interacting with NS3 to promote DENV replication [46,47]. It is mostly conserved among all dengue serotypes, making it an excellent target for antiviral development. Cordycepin an adenosine derivative is known to inhibit the NS5 MTase activity by binding to SAM-binding site and it is also found to bind to another domain of NS5 to inhibit RdRp activity of NS5 [46]. RK-0404678, a small molecule bound to NS5 and inhibited DENV replication. One binding site is in the thumb domain and the other sits in the active site. Binding of RK-0404678 to active site brings in a conformational change around Tyr607 residues [9]. The small molecules, C9 and C30, are found to inhibit the interaction between NS3 and NS5 by binding to the cavity B of NS5. This site was highly conserved all across the DENV serotypes resulting in showing efficacy of these compounds on all four serotypes. These small molecules were also found to extend their antiviral spectrum across other *Flaviviruses* like ZIKA and WNV [106]. Using a cell-based assay a study has shown that compound 29 binds to an allosteric binding pocked in the interface of thumb, palm subdomains of DENV RdRp and inhibit the DENV NS5 activity. This site was highly conserved across all the four serotypes leading to its efficacy on all four serotypes [112]. Leaf and bark extracts of plants belonging to *Myrtopsis corymbosa* of the *Rutaceae* family strongly inhibit the dengue NS5 RdRp activity. The compounds further extracted shown lesser efficacy in comparison to crude extracts indicating the antiviral properties of these compounds need other molecules from the crude extracts [49]. Direct acting anti-virals are summarized in Table 1.

**Table 1:** Direct acting anti-virals

Drug	Target(s)	Mechanism(s) of action	Reference
1662G07 and analogs	Envelope protein	Fusion inhibition	[50]
NITD448			[51]
1OAN1			[52]
Rolitetracycline			
Doxycycline			
A5			[53]
Compound 6			[54]
P02		Virus entry inhibition	[55]
EF		Block virus binding and inhibit entry	[56]
Geraniin			[57,58]
DET2			[59]
DET4			[59]
MLH40	PrM/M Protein	Block interaction between dengue M and E proteins	[60]
VGTI-A3			[61]
VGTI-A3-03			
Honeysuckle (*Lonicera japonica* Thunb.) extracts		Inhibition of viral replication and NS1 expression	[38]
Ivermectin	NS3 helicase and NS2B-NS3 protease	Inhibit the complex activity	[41,62]
ST-610	NS3 helicase	Helicase inhibitor	[63]
Suramin			[64]
Compound 25			[65]
Compound 7			[66]
Protegrin-1	NS 2B-NS3 complex	Protease inhibitor	[39]
Retrocyclin-1			[40]
Nelfinavir			[67]
Carnosine			[68]
Palmatine			[69]
Thiazolidinone-peptide hybrids			[70]
Compound 32			[71]
Compound 1			[72]
166347			[73]
ARDP0006			[74]
ARDP0009			
Compound 7n			[75]
Diaryl(thio)ethers			[76]
Compound C			[77]
Compound D			
Compound F (tolcapone)			
SK-12			[78]
Compound 104			[79]
Ltc1			[80]
BP13944			[81]
Policresulen			[82]
BP2109			[83]
MB21			[19]
Compound 45a			[84]
Compound 14			[85]
AM404	NS4B	Inhibition of NS4B activity	[44]
Compound 1a			[45]
Compound 14a			[45]
NITD-618			[17]
AZD0530			[31]
Dasatinib			
JNJ-1A			[86]
NITD-688			[87]
JNJ-A07		Block interaction between NS4B and NS3	[88]
Compound B	NS4A	Inhibit replication	[89]
Cordycepin	NS5	Inhibit viral replication by blocking viral RNA capping (MTase) activity of NS5	[48]
Azidothymidine-based triazoles			[90]
Compound 10			[18]
BG-323			[91]
NSC 12155			[92]
*Myrtopsis corymbose* extracts		Inhibit NS5 RdRp activity	[49]
RK-0404678			[9]
Trigocherrins			[93]
Trigocherriolides			[93]
Chartaceones			[94]
Avicularin			[95]
Quercitrin			
Betulinic acid			
Spiraeoside			
Rutin			
Pyridobenzothiazolones			[96]
(E)-tridec-2-en-4-ynedioic			[97]
Octadeca-9,11,13-triynoic acid			
Octadic-13-en-9,11-diynoic acid			
Octadic-13-en-11-ynoic acid			
C29			[98]
7-deaza-2′-C-methyl-adenosine		Viral replication inhibitor	[99]
INX-08189			[100]
BCX4430			[101]
Balapiravir			[102]
NITD008			[102]
2′-C-methylcytidine			[103]
C9 C30	NS3-NS5 complex	Block interaction between NS3 and NS5	[104]

## Small molecules targeting host proteins and signalling pathways

Besides, researchers developed small molecules targeting host factors like cellular receptors and signalling pathways, which are pro-viral for DENV replication. The major drawback of this approach is hindrance to the natural function of these targets in the host and increased cell cytotoxicity [105]. Bovine lactoferrin and carbohydrate-binding agents were found to inhibit the DENV entry in the DC-SIGN^+^ monocytes but not in DC-SIGN deficient monocytes indicating these compounds inhibit the virus entry by blocking the binding of DENV virus to DC-SIGN [106,107]. A linear PD1, CD44 peptide isolated from heparin sulphate receptor and a heparin sulphate mimic PG545 have shown an inhibitor effect on DENV entry in *in vitro* and *in vivo* experiments [108,109]. Studies have also shown that drugs like 3-MA and Ka-003 which inhibit autophagy has affected the viral replication, but other studies show that autophagy activators like rapamycin helps DENV propagation indicating host autophagy machinery has both anti-viral and pro-viral role in DENV replication [175,176]. Study from our lab has shown that a platelet chemokine released upon platelet activation during dengue infection acts as a proviral by binding to CXCR3 receptor on monocytes. Binding of PF4 to CXCR3 led to inhibition of IFN responses and autophagy resulting in enhanced viral replication. AMG487 a selective inhibitor of CXCR3 has reversed the effects of PF4 and rescued mice from dengue infection [1,33], but this drug was not passed through clinical trials due to its non-linear pharmacokinetics and efficacy problems. We further used computational studies and found a small molecule 7D which potently inhibits all four DENV serotypes in-vitro and DENV2 in in-vivo. So further exploration of host mechanisms involved in DENV replication are necessary to effectively manage the disease burden. The host-directed antiviral are summarized in (Table 2).

**Table 2:** Host directed anti-virals

Drug	Target(s)	Mechanism(s) of action	Reference
NITD-451	Translation machinery	Inhibition of viral RNA Translation	[110]
Narasin	Ionophore	Inhibit the release of viral RNA into cytoplasm	[111]
Lactimidomycin	Translation Machinery	Inhibition of viral RNA Translation	[112]
ST081006			[113]
Bromocriptine			[114]
Dasatinib	c-Src kinase	Inhibit dengue virion Assembly	[115]
Castanospermine	calnexin	Inhibit viral release	[116]
Brefeldin A	Protein Trafficking	Inhibit viral assembly, Maturation and release	[117]
Bovine lactoferrin	DC-SIGN receptor	Block the binding of DENV to DC-SIGN receptor	[106]
*Hippeastrum hybrid* (HHA)			
*Urtica dioica* (UDA)			
*Galanthus nivalis* (GNA)			
PD1 CD44	Heparan sulphate	Inhibit the interaction of dengue envelope protein to heparan sulphate	[108]
PG545			[109]
Fucoidan			[118]
PI-88			[119]
dl-galactan hybrid C2S-3			[120]
iota-carrageenan G3d			
CF-238			[121]
Sulfated galactomannan			[122]
Curdlan sulfate			[123]
Chondroitin sulfate E			[124]
P4 P7	β3 integrin	Inhibit the interaction between DENV and β3 integrin	[125]
AMG487	CXCR3 inhibitor	Inhibit PF4 interaction with CXCR3	[33]
3-MA	Autophagy machinery	Inhibit autophagy initiation	[175]
Ka-003		Block autophagolysosome formation	[176]

### Animal models for dengue used in antiviral and vaccine testing

The animal models of infectious diseases play a very important role in understanding the disease pathophysiology as well as development of therapeutics. Unlike other viral diseases, animal models for dengue have been crucial in understanding the pathogenesis, immune response, and testing potential treatments or vaccines. Additionally, animal models have provided valuable insights into the development of severe dengue disease, such as dengue haemorrhagic fever (DHF) and dengue shock syndrome (DSS) [126–128]. However, developing suitable animal models for DENV infection presents several challenges. Several animal models have been used in dengue infection research, including mouse models, non-human primate models, swine models, and shrew models [126,130–132]. Mouse models have played a crucial role in understanding pathogenesis and developing potential therapeutics and vaccines against the DENV. The best mouse model to employ in DENV infection and pathogenesis research has not yet been discovered [130,133]. AG129 mice, which are deficient in both IFN-α/β and IFN-γ receptors, have shown replication of DENV and dengue symptoms like thrombocytopenia, vascular leakage, and high viremia. AG129 mice infected with the adapted DENV strains provided a robust platform for testing therapeutic antibodies and other antiviral compounds [130–132,134–136]. Humanized mice, which are immunodeficient mice engrafted with human cells or tissues, have become invaluable tools for studying DENV infection includes, NSG (NOD/SCID/IL2rγ-/-) immunodeficient mice engrafting with human CD34^+^ hematopoietic stem cells (HSCs) and BLT Mice (Bone Marrow, Liver, Thymus) mice are developed by transplanting human fetal liver and thymus tissues under the kidney capsule of immunodeficient mice, followed by the injection of human HSCs [130–133]. Non-human primate (NHP) models have been pivotal in the preclinical testing of antiviral therapies and vaccines against DENV, owing to their genetic, physiological, and immunological similarities to humans. Rhesus macaques (Macaca mulatta) and cynomolgus macaques (Macaca fascicularis) are among the most commonly used NHP species for dengue research. Studies evaluating antiviral therapies and candidate vaccines, including live attenuated, inactivated, and recombinant vaccines, have been tested in these models to assess safety, immunogenicity, and protective efficacy [130,132,137–140]. Swine have been identified as an important animal model for dengue infection research due to their physiological similarities to humans. Recent studies have demonstrated that a specific strain of Yucatan miniature pig, Sus scrofa, exhibits physiological and immunological responses closely mirroring those observed in humans. Moreover, swine models have been used to evaluate vaccine candidates and antiviral therapies against DENV [132,133,141,142]. The tree shrew (Tupaia belangeri) has emerged as an alternative animal model for studying DENV infection, offering unique advantages in dengue research. Tree shrews are susceptible to all DENV serotypes infection, showing signs of viremia and clinical symptoms similar to human dengue, including fever, thrombocytopenia, and vascular leakage [143–145]. The model has been used to study the dynamics of immune response to DENV, including the role of T cells, B cells, and cytokines in viral clearance and disease resolution. Studies testing antiviral therapies in tree shrews have demonstrated the efficacy of treatments in reducing viral load and improving clinical outcomes [132,133,147,148].

## Preclinical and clinical studies of small molecule antivirals against dengue

Preclinical trials are the important steps to understand the underlying mechanisms of a disease and also evaluating the potential efficacy of interventions. Although very few, compounds like geraniin, PG545, NITD-622 and HS-1 have been in the preclinical trial pipeline against dengue. Small animals and non-human primates have been utilized in these trial against dengue [149–152]. Besides, clinical trials for dengue using several drugs are in progress. Importantly, close monitoring of results and analysis of comprehensive data are the crucial steps of a clinical trial towards determining the better efficacy and safety of a drug in human.

### Ivermectin

Ivermectin, an antiparasitic medication, has attracted interest for its potential antiviral properties, including its efficacy against all four serotypes of the DENV (DENV1-4). It inhibits the nuclear transport of viral proteins by binding to and inhibiting the importin α/β1 heterodimer, which disrupts the replication cycle of DENV by preventing the nuclear localization of dengue NS5, an RNA-dependent RNA polymerase (RdRp). Studies have shown that ivermectin significantly reduces viral RNA levels and the production of infectious virions in infected cell cultures. Clinical trials, such as the phase I/II trial (NCT02045069), have explored ivermectin as a dengue treatment, focusing on safety and efficacy. Participants received various doses over two or three days, with outcomes measuring the resolution of viremia, NS1 antigen clearance, and fever reduction. Initial results were promising in reducing NS1 protein levels, which are linked to dengue severity, but the trial did not meet its primary endpoint due to fluctuating dengue incidence rates during enrolment. While the clinical benefits of ivermectin for dengue remain inconclusive, ongoing studies are investigating its pharmacokinetics and higher doses to determine if they can effectively inhibit DENV replication and improve clinical outcomes [155–160] preclinical and clinical data for drugs against dengue virus are shown in (Table 3).

**Table 3:** Comprehensive table including preclinical and clinical data for drugs against DENV

Drug and type	Stage	Mechanism of action	Preclinical data	Clinical data	References
Remdesivir Antiviral	Preclinical	Inhibit RNA-dependent RNA polymerase (RdRp)	Inhibited DENV replication in cell culture and a significant reduction in viral load improves the survival rate of mice. Reduced viremia and improved clinical outcomes following dengue infection in NHP	The study focused on safety, tolerability, and pharmacokinetics in healthy volunteers. It also evaluated efficacy in NS1-positive dengue patients; primary endpoints included a reduction in viral load and symptom improvement	[153,154]
Ivermectin Antiparasitic (repurposed)	Phase 2	Inhibits nuclear import of viral proteins	Demonstrated antiviral activity *in vitro* against DENV	Mixed results in clinical trials; issues with dosage and bioavailability	[151,156–159]
Zanamivir Antiviral (Influenza)	Phase 2 (ZAP-Dengue Trial)	Neuraminidase inhibitor	Limited preclinical data for dengue; primarily used for influenza	Investigated for severe dengue with vascular permeability syndrome in Phase 2 trial	[161,162]
Niclosamide Anthelmintic (repurposed)	Preclinical/Phase 1	Disrupts viral replication and host cell pathways	Effective against DENV *in vitro*; challenges with bioavailability	Early Phase 1 trials were initiated to assess safety and tolerability	[163–165]
JNJ-64281802 (Direct-acting antiviral)	Phase 2	Inhibits NS3-NS4B protein interaction	Effective in reducing viral load in non-human primates and mice. Potent activity against multiple dengue serotypes in animal models	Phase 2 trials underway to determine efficacy and safety in humans	[32,166,97]
AT-752 (Direct-acting antiviral)	Phase 1/2	Inhibits viral replication	Preclinical studies showed a significant reduction in viral load in animal models	Ongoing Phase 1/2 trials assessing safety, tolerability, and preliminary efficacy	[167,168]
Favipiravir Broad-spectrum antiviral	Phase 2/3	Inhibits viral RNA polymerase	Demonstrated broad antiviral activity including against dengue *in vitro* and in animal models	Mixed results in reducing viral load and symptoms in Phase 2/3 trials	[169,170]
Balapiravir Direct-acting antiviral	Phase 2	Inhibits viral RNA polymerase	Initially promising* in vitro* results; limited efficacy in animal models	Phase 2 trials showed mixed results, leading to discontinuation of further development	[171]
CelgosivirHost-directed antiviral	Phase 2	Inhibits host α-glucosidase, disrupting viral glycoprotein processing |	Shown to reduce viral replication in preclinical studies	Phase 2 trials demonstrated some efficacy; potential for combination therapy	[172]
Vitamin D	Phase 2	Immunomodulatory	Modulating immune response and up-regulates the antimicrobial peptides and cytokines, which could potentially reduce the severity of dengue infection. Laboratory studies have suggested that Vitamin D3 can inhibit the replication of DENV in cell cultures	Ongoing clinical trials are evaluating the efficacy of high-dose Vitamin D3 in patients with dengue fever. Participants receive doses of 200,000 IU or 400,000 IU Vitamin D3 orally to assess its impact on disease progression and severity	[173,174]

### Zanamivir

Zanamivir is an antiviral drug primarily used for treating and preventing influenza by inhibiting the neuraminidase enzyme essential for viral replication and release. Although it is effective against influenza, zanamivir is not inherently effective against the DENV, which lacks neuraminidase. Consequently, zanamivir’s direct mechanism of action does not apply to DENV. However, preclinical studies have shown that zanamivir can reduce vascular leakage caused by dengue in mice, addressing a major cause of death in severe dengue infections [164]. Zanamivir is currently being investigated in a phase I clinical trial (NCT04597437) named ZAP-DENGUE, conducted by George Washington University and other institutions. This randomized, double-blind, placebo-controlled trial aims to evaluate the safety and efficacy of intravenous zanamivir for treating vascular permeability syndrome in severe dengue cases. The trial, involving 74 participants with dengue fever, started in March 2024 and is expected to be completed by September 2025. Primary outcomes include treatment-emergent adverse events and levels of endothelial glycocalyx biomarkers, which indicate vascular damage [161,162].

### Niclosamide-based antiviral

Niclosamide, an anthelmintic drug approved for the treatment of tapeworm infections, has gained attention for its potential repurposing as a broad-spectrum antiviral agent. Researchers are exploring niclosamide’s efficacy against various viral infections, including DENV, due to its ability to inhibit viral replication and modulate host cell pathways. *In vitro* studies have demonstrated that niclosamide effectively inhibits the replication of DENV in cell cultures. It reduces viral RNA levels and the production of infectious virions. Niclosamide inhibits DENV infection by interfering with endosomal acidification, thereby preventing viral entry and replication within host cells, independent of mTOR signalling [163,164]. Additionally, preclinical studies in animal models have shown that niclosamide can decrease viremia and improve survival rates in dengue-infected subjects. Hyundai Bioscience is conducting clinical trials in Brazil, a region with a high burden of dengue. These trials aim to evaluate the effectiveness of a niclosamide-based antiviral formulation in reducing the severity of dengue symptoms and viral load. Niclosamide-based antivirals represent a promising avenue for dengue treatment, supported by preclinical efficacy and ongoing clinical trials. If proven effective, niclosamide could become an important tool in managing dengue, particularly in regions with high incidence rates. Continued research and clinical validation are essential to fully establish its therapeutic potential and address the challenges associated with its use [164,165].

### JNJ-1802

Is an innovative antiviral compound shows significant promise against the DENV. This small molecule inhibits the DENV replication by blocking the interaction between the non-structural proteins NS3 and NS4B, which are critical for viral replication. This compound has shown strong efficacy in preclinical studies, demonstrating protection in non-human primates and mice against DENV serotypes DENV-1 and DENV-2 and effectiveness against all four dengue serotypes in mouse models with a favourable safety profile. In a Phase 1 clinical trial, JNJ-1802 was found to be safe and well-tolerated in humans. In a recent human challenge study, JNJ-1802 exhibited a dose-dependent antiviral effect against DENV serotype 3 (DENV-3), reducing the detectability of viral RNA and delaying the onset of detectable viremia [32,97,166 ]. These findings are encouraging as J&J continues to conduct a large-scale phase 2 trial (NCT05048875) involving 1,850 participants across several countries where dengue is endemic, such as Brazil, Colombia, and Thailand. Overall, JNJ-1802 represents a significant advancement in the fight against dengue, offering potential both as a prophylactic and therapeutic agent. If successful, it could greatly impact global health by providing a much-needed antiviral treatment for dengue, which affects millions of people annually and currently lacks specific therapeutic options. These trials are critical for understanding the safety, efficacy, and optimal dosing of JNJ-64281802 in combating dengue virus infections.

## Conclusion and outlook

Dengue is an endemic with frequent outbreaks across the globe with significant disease burden due to global warming, environmental changes, pollution, rapid urbanization and increasing population. WHO has taken up the goal of decreasing the dengue related mortality and morbidity of dengue by 2020. Therefore, there is an urgent need for the countries to further increase the collaborations and also make the research results available for the peers in the field of dengue research. Research should be intensifying in the aspects of disease pathogenesis to better understand the course of pathological events in case of dengue.

Though multiple small molecules being reported to be having antiviral activity against the DENV, very few of them were able to be pursued further for clinical trials. This is due to lack of the complete understanding of the dengue disease pathogenesis. There is significant involvement of government agenesis along with private research institutes and medical professionals during COVID-19 pandemic in development of treatment regimens and vaccines. Researchers should collaborate with the medical professionals in better understanding the pathological events during the course of dengue fever. This will help in exploring the causes of disease with new perspectives. Due to unclear disease pathogenesis, till date there was no specific small molecule for clinical use is still lacking. The major setbacks for this also include difficulties in establishing an efficient screening platform for dengue anti-virals, development of pre-clinical animal models for drug efficacy testing and discovery of new strategies in development of small molecules against dengue. Therefore, there is an urgent need in understanding the dengue disease pathogenesis which help in development of preclinical animal models and discovering new target molecules against which small molecules can be designed and developed.

## Abbreviations

DC: dendritic cell
DENV: dengue virus
DENV-3: dengue virus serotype 3
DHF: dengue haemorrhagic fever
DSS: dengue shock syndrome
EGFR: epidermal growth factor receptor
ER: endoplasmic reticulum
ERK: epidermal growth factor receptor-related kinase
HSC: hematopoietic stem cell
ISG: interferon-stimulated gene
NHP: non-human primate
